# Safety and Cost-Savings of Same-Day Discharge Trans-Radial Percutaneous Coronary Intervention in Trinidad and Tobago

**DOI:** 10.7759/cureus.9568

**Published:** 2020-08-05

**Authors:** Richard Ramsingh, Dale Maharaj, Gianni Angelini, Risshi D Rampersad

**Affiliations:** 1 Cardiovascular Disease, Caribbean Heart Care Medcorp, Port-of-Spain, TTO; 2 Vascular Surgery, Caribbean Vascular & Vein Clinic, Port-of-Spain, TTO; 3 Cardiac Surgery, Bristol Heart Institute, Bristol University, Bristol, GBR; 4 Cardiology, Caribbean Heart Care Medcorp, Port-of-Spain, TTO

**Keywords:** same day discharge, trans-radial, pci, small island developing states, trinidad and tobago, developing country

## Abstract

Introduction: Same-day discharge percutaneous coronary interventions (SDD-PCI) may be quite impactful on healthcare burden for small island developing states (SIDS) such as Trinidad and Tobago.

Methods: From June 2012 to November 2014, 11 patients underwent SDD trans-radial PCI and followed up at one-month and three months. Data was retrospectively reviewed from a prospectively entered unit-maintained cardiology database. Baseline patient characteristics, in-hospital expenditure, and complications were assessed. Descriptive statistical analysis was performed in Microsoft Excel.

Results: The mean age at SDD-PCI was 50.90±9.96 and nine were male. Nine patients were of East Indian Caribbean ethnicity. Six were diabetic and five were hypertensive. Procedural success was 100% with no major early complication or three-months complications; patient satisfaction was achieved with a potential in-hospital savings up to $1480 USD per patient.

Conclusion: This SDD approach for elective trans-radial PCI may be safe and cost-effective in properly selected patients and merits a review of relevant policy issues in Trinidad and Tobago.

## Introduction

The increasing prevalence of coronary artery disease has led, in recent years, to growing number of percutaneous coronary interventions (PCI) worldwide [1]. However, PCI admissions represent a significant burden on the available resources and challenges healthcare systems to reduce costs of delivery without compromising access and safety [2-4]. Same-day discharge (SDD) PCI has the potential for reducing costs and achieving patient satisfaction while maintaining clinical effectiveness. However, whether SDD-PCIs can be delivered safely, without compromising clinical outcomes, is unknown in the Caribbean. The present study aims to describe the safety and cost-savings of SDD elective trans-radial PCI in a selected cohort of stable angina and non-ST-elevation acute coronary syndrome (ACS) patients.

## Materials and methods

A retrospective study of prospectively collected data was conducted from June 2012 to November 2014 at St. Clair Medical Centre, Caribbean Heart Care Medcorp. The study was conducted in accordance with the principles of the Declaration of Helsinki. The local audit committee approved the study, and the requirement for individual patient consent was waived. The cardiology unit is located in Port of Spain, Trinidad and Tobago and serves a catchment area of 500,000 population. Each patient potentially dischargeable the day of their PCI was prospectively identified. Criteria included elective PCI, low-risk type A lesion, and living one hour away from the hospital. In total, 11 elective PCI patients were selected based on these criteria. Reasons for exclusion included inadequate home support, procedural complexity, medium to high-risk coronary lesions, left ventricular dysfunction, ST-elevation myocardial infarction (STEMI), and multiple co-morbidities. All patients were premedicated with ASA and clopidogrel prior to PCI. The trans-radial approach was used for PCI. Procedural success was defined as technical success with no in-hospital major adverse cardiac events (MACE). Following the procedure, patients were monitored for at least six hours before being seen by a cardiologist at which time they received standardized discharge instructions including an emergency contact number and close monitoring by caregiver(s). Complications were defined as death, acute vessel closure, revascularization, Q-wave myocardial infarction, stroke, bleeding, or hematoma. Patient satisfaction was assessed the next day as a binary “yes or no” option. Cost savings were calculated as the difference of cost to an SDD-PCI patient from a control comparable non-SDD-PCI patient - whose average length-of-stay was one day overnight. Total cost savings were calculated as a product of estimated cost savings and number of patients.

Patient follow-up was done the next day which included duplex ultrasonography of the radial artery. Clinic visits at one and three months assessed post-operative complications, and need for further investigations.

## Results

Eleven patients qualified for SDD elective PCI prospectively (patient pre-operative characteristics are summarized in Table 1). There were nine male and two female patients with an age range of 29-67 years (mean 50.90±9.96 SD), nine patients of East Indian descent, and two of Afro-Caribbean descent. Indications for elective PCI included five stable angina (56%), three unstable angina (27%), two non-ST elevation myocardial infarction (18%). Asymptomatic patients accounted for 9% of patients. The mean left ventricular ejection fraction was 51.80±8.71.

**Table 1:** Baseline Characteristics of Patients Who Underwent Same-Day Discharge Trans-Radial Elective PCI Values are means±SD. PCI: percutaneous coronary intervention; CABG: coronary artery bypass graft; CAD: coronary artery disease

Characteristic	All Patients
Age – yrs	50.90±9.96
Male sex – no./total no.	9/11
East Indian ethnic group – no./total no.	9/11
Afro-Caribbean – no./total no.	2/11
Body-mass index	27.26±2.82
Diabetes – no./total no.	6/11
Hypertension – no./total no.	5/11
Previous Myocardial Infarction – no./total no.	7/11
Family History of CAD – no./total no.	3/11
Smoker – no./total no.	2/11
Previous PCI – no./total no.	1/11
Previous CABG – no./total no.	1/11
Left ventricular ejection fraction -%	51.80±8.71

The culprit lesion was located in the left anterior descending coronary artery (7, 64%), right coronary artery (2, 18%), and circumflex artery (2, 18%). They were all stented with drug-eluting stents. Procedural success was 100% with no complications of death, acute vessel closure, Q-wave myocardial infarction, stroke, bleeding, or hematoma. All patients were discharged on the same day. Radial artery patency rate assessed with duplex ultrasound by a trained Registered Physician in Vascular Interpretation (RPVI) during the next day visit showed 100% patency with good triplane flow in all cases. On day one of the follow-up interview, all patients expressed satisfaction with SDD.

At one- and three-months follow-up, there were no adverse events - myocardial infarction, stroke, bleeding, hematoma, chest pain, emergency room visits, re-hospitalization - reported after PCI. Furthermore, no patients experienced chest discomfort, and none had sought medical assessment. The estimated potential savings with SDD-PCI ranged from $890 - $1480 USD per patient with a total potential savings of $9790-$16280 USD. 

## Discussion

This series of patients undergoing SDD trans-radial elective PCI demonstrates feasibility and safety in a group of selected patients at low risk for post-discharge complications. The main findings included, no major early and three months complications, patient satisfaction, and a potential in hospital savings up to $1480 USD per patient.

SDD specifically refers to a patient who presents for elective PCI, undergoes the procedure, and after a period of supervised recovery is sent home on the same calendar day [5]. Understandably, there is primary medico-legal concern surrounding length of stay (LOS) after PCI pertaining to the occurrence of complications that may manifest after discharge. In 2018, SDD-PCI was endorsed by the Society for Cardiovascular Angiography and Interventions (SCAI) in their updated 2018 Expert Consensus Document on LOS following PCI as a safe and reasonable approach for certain PCI patients [5]. Criteria included event monitoring 4 to 6 hours after the completion of elective PCI, stable patient, a successful procedure, adequate hemostasis of the access site, and strong social supports to follow through with post-PCI instructions for care [5]. In keeping with advancements in vascular access techniques, trans-radial access was used as the preferred method for elective PCI as it was demonstrated to be safe and comfortable compared to trans-femoral access, both locally and internationally [2,5,6]. In the event of an overnight admission after PCI due to concerns of peri-procedural events, an on-call interventional cardiologist was present. According to a retrospective study of more than 2100 trans-radial PCIs, Small et al. found most non-target vessel complications to occur either within the first 6 hours (3.4%) and target vessel closures to occur after the 24th hour (1.9%), with none occurring in the monitoring period afforded by overnight admission (hours 6-24) [7]. Notably, there were no complications arising during the six-hour monitoring period and therefore no transitions from SDD to overnight stay. This may be attributed to strict exclusion criteria and adherence to anti-platelet medication after discharge.

Trinidad and Tobago is a small twin-island state comprising a population of 1.39 million people. With the high economic burden of non-communicable diseases (NCDs), appropriate funding of the health sector in this small island developing state is a major step towards tending to the needs of the population. Public health services are currently free for patients, however, there has been a multitude of reports highlighting the poor performance of the public health sector in large part due to overcrowding [8,9]. This has been attributed to poor hospital throughput - defined as the number of patients served in a unit of time [8]. With the increased demand for hospital beds and high volume of angiograms and PCIs, hospitals remain under constant pressure to provide patients with appropriate post-procedural care and safe discharge. A proposed solution to this increased need is to shorten the LOS and reduce the need for bed usage. SDD-PCI potentially opens beds for other inpatient admissions in hospitals operating at near maximal capacity. Therefore, there is potential to reduce length-of-stay cost, overnight stays allowing for greater bed-flow efficiency, and potentially improve patient satisfaction on a larger scale.

Regarding cost-savings, in Canada, roughly $1200 Canadian dollars ($915 USD) are saved per patient with SDD-PCI and in the United States, more than $5000 USD [2,4]. These savings are driven by reduced supply requirements, and room and board costs [4]. In our series, savings amounted to $890 -$1480 USD per patient. The rate of PCI is currently unknown in Trinidad, however, there was approximately $1.88 million USD spent on non-SDD-PCI in 2009 ($2M USD adjusted for inflation) [10]. We expect that significant cost savings would be made by adopting the practice of SDD-PCI in selected patients.

With regard to patient satisfaction, Kim et al. in 2013 conducted a randomized controlled trial that found many patients prefer to recover from a PCI procedure at home rather than spend a night in the hospital [11]. This observation, well established in same-day surgery, was also made in our series [12].

There are some limitations in our study including its small sample size, the strict criteria of inclusion, and the fact that it was conducted in a single hospital. However, there are few cardiac catheterization centres in Trinidad routinely performing trans-radial PCI. We undertook this study to assess the feasibility and safety of the procedure. Nonetheless, our work is consistent with our international counterparts who have showed that highly selected SDD-PCI patients had no difference in clinical outcomes at 30 days and had lower rates of clinical complications [2-4].

Discharge planning after PCI is ultimately a medical decision and should be based on what is the best care for the patient. While cost-savings should not affect discharge decisions, the financial impact and bed-flow efficiency of LOS decisions merits a review of the relevant policy issues.

## Conclusions

SDD for elective trans-radial PCI may be a safe and cost-effective management plan in properly selected patients in Trinidad and Tobago. Further studies are required to expand this approach to more complex cases.
